# A high burden of asymptomatic genital tract infections undermines the syndromic management approach among adolescents and young adults in South Africa: implications for HIV prevention efforts

**DOI:** 10.1186/s12879-018-3380-6

**Published:** 2018-10-03

**Authors:** Angela Kaida, Janan J. Dietrich, Fatima Laher, Mags Beksinska, Manjeetha Jaggernath, Megan Bardsley, Patricia Smith, Laura Cotton, Pooja Chitneni, Kalysha Closson, David A. Lewis, Jenni A. Smit, Thumbi Ndung’u, Mark Brockman, Glenda Gray

**Affiliations:** 1 Faculty of Health Sciences, Simon Fraser University, Blusson Hall Rm 10522, 8888 University Drive, Burnaby, B.C. V5A 1S6 Canada; 2 Perinatal HIV Research Unit (PHRU), Faculty of Health Sciences, University of the Witwatersrand, Johannesburg, South Africa; 3 Maternal Adolescent and Child Health (MatCH) Research Unit (MRU), Faculty of Health Sciences, University of the Witwatersrand, Durban, South Africa; 4 London School of Hygiene and Tropical Medicine, London, UK; 5 Harvard combined Infectious Diseases Fellowship, Boston, MA USA; 6 Centre for HIV and STIs, National Institute for Communicable Diseases, Johannesburg, South Africa; 7 Faculty of Medicine and Health & Marie Bashir Institute for Infectious Diseases and Biosecurity, University of Sydney, Sydney, Australia; 8 HIV Pathogenesis Programme and Africa Health Research Institute, University of KwaZulu-Natal, Durban, South Africa; 9 Ragon Institute of Massachusetts General Hospital, Massachusetts Institute of Technology and Harvard University, Cambridge, MA USA; 10 Max Planck Institute for Infection Biology, Berlin, Germany; 11 South African Medical Research Council, Cape Town, South Africa

**Keywords:** Adolescents and young adults, Youth, Women, Sexually transmitted infections, Genital tract infections, HIV prevention, Screening, Syndromic management, Performance analysis, South Africa

## Abstract

**Background:**

Youth in southern Africa, particularly adolescent girls and young women, are a key population for HIV prevention interventions. Untreated genital tract infections (GTIs) increase both HIV transmission and acquisition risks. South African GTI treatment guidelines employ syndromic management, which relies on individuals to report GTI signs and symptoms. Syndromic management may, however, underestimate cases, particularly among youth. We compared genital tract infection (GTI) prevalence by symptom-based and laboratory assessment among sexually-experienced youth in South Africa, overall and stratified by sex.

**Methods:**

Interviewer-administered surveys assessed socio-demographics, behaviors, and GTI symptoms among 352 youth (16-24 yrs., HIV-negative or unknown HIV status at enrollment) enrolled in community-based cohorts in Durban and Soweto (2014–2016). Laboratory tests assessed HIV, *Chlamydia trachomatis* (CT), *Neisseria gonorrhoeae* (NG), *Mycoplasma genitalium* (MG), *Trichomonas vaginalis* (TV) infections and, among females, bacterial vaginosis (BV) and Candida species. Youth with genital ulcers were tested for HSV-2 and syphilis. We assessed sensitivity (and specificity) of symptom-based reporting in identifying laboratory-confirmed GTIs.

**Results:**

At baseline, 16.2% of females (32/198) and < 1% (1/154) of males reported ≥1 GTI symptom. However, laboratory tests identified ≥1 GTI in 70.2% and 10.4%, respectively. Female CT prevalence was 18.2%, NG 7.1%, MG 9.6%, TV 8.1%, and 5.1% were newly diagnosed with HIV. BV prevalence was 53.0% and candidiasis 9.6%. One female case of herpes was identified (0 syphilis). Male CT prevalence was 7.8%, NG 1.3%, MG 3.3%, TV < 1%, and 2.0% were newly diagnosed with HIV. Overall, 77.8% of females and 100% of males with laboratory-diagnosed GTIs reported no symptoms or were asymptomatic. Sensitivity (and specificity) of symptom-based reporting was 14% (97%) among females and 0% (99%) among males.

**Conclusion:**

A high prevalence of asymptomatic GTIs and very poor sensitivity of symptom-based reporting undermines the applicability of syndromic GTI management, thus compromising GTI control and HIV prevention efforts among youth. Syndromic GTI management does not meet the sexual health needs of young people. Policy changes incorporating innovations in GTI diagnostic testing are needed to reduce GTIs and HIV-associated risks among youth.

**Electronic supplementary material:**

The online version of this article (10.1186/s12879-018-3380-6) contains supplementary material, which is available to authorized users.

## Background

Adolescents and young adults (aged 15–24 years) account for one-third of incident adult HIV infections in South Africa. Girls and young women are particularly at risk, with nearly 2000 young women acquiring HIV every week [1, 2]. Accordingly, reducing HIV risk among youth remains a pressing global public health priority [3].

Untreated genital tract infections (GTIs) increase risk of HIV acquisition and transmission through several organism-specific pathways [4, 5]. Untreated HIV can also increase GTI severity and duration through immune suppression pathways. For women, untreated GTIs are a significant cause of reproductive morbidity, including pelvic inflammatory disease, tubal factor infertility, adverse birth outcomes, and infertility [6].

The success of GTI control programs is predicated on timely and effective diagnosis and treatment [7]. South African GTI treatment guidelines employ syndromic management [8], consistent with WHO recommendations [9]. Syndromic management is based on the identification of syndromes (i.e., consistent groups of symptoms and clinical signs) associated with infection by a defined pathogen, followed by syndromic treatment for the most common causative GTI organisms.

While syndromic GTI management can be used to treat and manage symptomatic GTIs at the primary healthcare level without the need for laboratory facilities or diagnostic tests, such an approach can underestimate GTI cases, particularly among women for whom an estimated 50–75% of those with a GTI are asymptomatic [10, 11]. Few studies from high HIV-burden settings have examined GTI prevalence or diagnostic performance of syndromic management among adolescents and young adults [11]. This is concerning given the dual burdens and synergies between HIV and GTIs among youth and the potential for long-term sequelae. There are also concerns about the ability of syndromic treatment to differentiate between the etiologies of the disease, leading to the potential for over-prescription of antibiotics and attendant risks of antimicrobial resistance [5].

This analysis measured and compared GTI prevalence by syndromic-based and laboratory assessment among sexually-experienced South African adolescents and young adults, stratified by sex. We assessed the performance of syndromic GTI management compared with the ‘gold standard’ of laboratory diagnosis (including measurement of sensitivity, specificity, positive predictive value [PPV], and negative predictive value [NPV]. In pursuing these objectives, we seek to inform GTI control programs for youth in the context of emerging biomedical HIV prevention efforts.

## Methods

### Study design and participants

We used baseline data from adolescents and young adults (females and males) enrolled in the AYAZAZI study, a youth-centered, dual-site, community-based, prospective cohort study focused on understanding linked patterns of socio-behavioural and biomedical HIV risk among youth in South Africa [12].

AYAZAZI study inclusion criteria included being 16–24 years of age, residing in Soweto or Durban, self-reporting an HIV-negative or unknown HIV status, and being willing and able to provide voluntary written informed consent. Exclusion criteria included current participation in another clinical or observational HIV prevention study. The Soweto cohort was based at the Perinatal HIV Research Unit (PHRU) located at Chris Hani Baragwanath Hospital. The Durban cohort was based at the Commercial City research site led by MatCH Research Unit (MRU).

We used posters, pamphlets, word-of-mouth, and in-person community outreach to recruit 425 participants (253 females, 172 males) across both sites. In Soweto, participants were also recruited through the PHRU’s HIV testing and counselling clinic while in Durban, participants were also recruited through a public sector reproductive health clinic at Commercial City. Enrolment occurred between November 2014 and April 2015 in Soweto and between September 2015 and April 2016 in Durban.

### Study procedures

Participants completed a structured questionnaire (supported by DataFAX™ software) at enrolment, administered in-person by youth interviewers. The questionnaire assessed demographic characteristics, social determinants of health, healthcare-seeking behaviours, sexual behaviors, pregnancy history, substance use, experiences of violence, and self-perceived HIV risk. Questionnaires were conducted in English, isiZulu, or Sesotho, as per participant preference. Participants received a 150 ZAR (~$12 USD) reimbursement per visit to compensate for transportation costs and time.

Presence of GTI symptoms was assessed via a nurse-administered questionnaire. All participants underwent a nurse-led physical examination (including genital examination for presence of a genital ulcer), consistent with standard treatment guidelines [8].

Urine (males) and nurse-collected vaginal swab (females) specimens were collected and transported daily to the National Institute for Communicable Diseases in Johannesburg (for the Soweto site) and the Global Clinical and Viral Laboratory (for the Durban site) for analysis. Using multiplex nucleic acid amplification test (NAAT) methods, all participants were tested for *Chlamydia trachomatis* (CT)*, Neisseria gonorrhoeae* (NG)*, Mycoplasma genitalium* (MG), and *Trichomonas vaginalis* (TV)*.* All females were tested for the presence of bacterial vaginosis (BV) and *Candida* species (CA) using Gram-stain techniques. Vaginal microbiota specimens were evaluated using Nugent criteria, which differentiate between BV (scores 7–10), intermediate microbiota (scores 4–6), and normal microbiota (scores 0–3) [13]. Participants with active genital ulcers identified during physical exams were tested for herpes simplex virus type 2 (HSV-2), *Treponema pallidum*, *Haemophilus ducreyi* and *C.trachomatis serovar L1-L3* via multiplex PCR using the Roche LightCycler. AYAZAZI clinical staff offered counselling and treatment (either onsite or via referral to a local clinic) to participants immediately following a syndromic diagnosis or after receipt of positive GTI laboratory results. Recent data demonstrating increased inflammation and thus HIV acquisition risk among women with asymptomatic BV informed study procedures to include asymptomatic BV cases as requiring treatment, after confirmation using Nugent criteria [14].

For female participants, research nurses performed rapid pregnancy testing using the QuickVue One-Step HCG urine test (Quidel Corporation, San Diego, CA, USA). Vaginal swabs were deferred for a week for menstruating women, and deferred until pregnancy resolution.

All participants were tested for HIV using the Abon™ HIV 1/2/0 Tri-Line HIV RDT test (Abon Biopharm Co. Ltd., Hangzhou, China) with confirmatory testing using the First Response™ HIV-1-2.0 Rapid Whole Blood Test kit (Premier Medical Corporation Private Ltd., Gujarat, India). Those who tested negative on the first rapid test were considered HIV-negative and offered standard HIV prevention counselling. Individuals who tested positive using both the rapid HIV tests were considered HIV-positive, counselled, and referred to a local clinic for further assessment, including CD4 testing, and counselling on initiation of antiretroviral therapy (ART). CD4 T cells were enumerated from whole blood using Tru-Count technology and analysed on a FACSCalibur flow cytometer (Becton Dickinson). If one rapid test was positive and the other negative, offsite confirmation was conducted using 3rd generation (i.e., antibody only) ELISA tests (MICROLISA HIV test, J.Mitra & Co. Ltd., New Delhi, India [Soweto]; Vironostika HIV Uni-Form II Plus O test, Biomerieux, Marcy L’Etoile, France [Durban]).

### Measures

We measured the prevalence and types of nine self-reported GTI symptoms (for both female and male participants) and associated seven syndromes, stratified by sex [8, 9] (Additional file 1: Table S1).

We measured the prevalence of laboratory-confirmed GTIs, stratified by sex. Ulcerative STIs were analysed separately from non-ulcerative STIs, as only the etiological distribution of ulcerative STIs could be reported as opposed to prevalence. We measured the prevalence of (a) each GTI screened, (b) ≥1 GTI, (c) co-infection with ≥2 non-ulcerative STIs, (d) co-infection with ≥1 non-ulcerative STI and BV, and (e) newly-diagnosed HIV.

### Statistical analysis

Of 425 participants enrolled in AYAZAZI, this GTI analysis was restricted to sexually-experienced (excluded *n* = 64) and non-pregnant (excluded *n* = 9) youth, yielding a final analytic sample of 352 (82.4% of the total cohort).

Descriptive statistics (median [IQR] for continuous variables and n [%] for categorical variables) were used to characterize baseline distributions of study variables, stratified by natal sex (female or male). Baseline differences between females and males were compared using Wilcoxon rank sum test for continuous variables and Pearson χ^2^ or Fisher’s exact test for categorical variables. Descriptive statistics were used to characterize (1) distribution of GTI symptoms and syndromes by sex and by study site; and (2) distribution of laboratory-confirmed infections by sex and study site**.**

For sensitivity, specificity, PPV, and NPV calculations [15], the gold-standard was a positive laboratory test result for any aetiological agent of the syndrome, defined as follows [8, 9]: Vaginal Discharge Syndrome (VDS): NG, CT*,* MG (cervical infections), or TV, BV, or CA (vaginal infections); Lower Abdominal Pain (LAP): NG, CT, or, MG*;* Male Urethritis Syndrome (MUS): NG, CT, MG, or TV; and Scrotal Swelling (SSW): NG or CT.

Sensitivity analysis of VDS was stratified by cervical, vaginal, and BV as the causative agent to compare performance statistics for each infection type and considering that the high BV prevalence could augment performance statistics relative to other VDS-associated organisms. Performance analysis of Genital Ulcer Disease (GUD) was not possible as only those with genital ulcers (*n* = 3) were tested for ulcerative-STIs. Data were analyzed with SAS version 9.1.

All participants aged 18–24 years provided voluntary informed consent at enrollment. For participants aged 16–17 years, parents/legal guardians provided voluntary written informed consent and the participant provided voluntary written informed assent. Ethical approval was provided by the Research Ethics Boards of Simon Fraser University (Canada), the University of the Witwatersrand (South Africa), and the University of KwaZulu-Natal (South Africa).

## Results

### Participant characteristics

Of 352 youth included in this analysis, 56% were female, 17.1% were less than 18 years old, 6.8% identified as LGBTQ (lesbian, gay, bisexual, transgender, and queer), 66.1% were currently in school and 25.9% reported high food insecurity. All participants identified as cis gender. Over one-quarter reported living in informal housing (27.6%) or having a personal monthly income < 400 ZAR (~$30 USD) (26.4%), with significant differences by sex (*p* < 0.05).

We also observed several differences in sexual and reproductive experiences by sex. While 55.6% of youth self-perceived as having no or low risk of HIV, 20.7% of females and 45.5% of males reported ≥2 sexual partners in the past 6 months (*p* < 0.001), and 80.6% of females and 51.3% of males reported inconsistent condom use (*p* < 0.001). A higher proportion of males than females reported ever having engaged (i.e., provided or received) in transactional sex (22.9% and 7.2%; respectively), while a higher proportion of females than males reported an age-disparate sex partner (i.e., ≥ 5 years older or younger) (34.9% and 12.4%; respectively) (Table 1).

**Table 1:** Baseline characteristics of sexually-experienced and non-pregnant youth (aged 16–24 years) enrolled in the AYAZAZI study in South Africa, stratified by sex (*n* = 352)

Characteristics	Overall *n* = 352 *n* (%)	Total *n*	Females *n* = 198	Males *n* = 154	*P*-value
			*n* (%)		
Socio-demographics					
Study Site		352			0.314
Soweto	173 (49.2)		102 (51.5)	71 (46.1)	
Durban	179 (50.8)		96 (48.5)	83 (53.9)	
Age category, years		352			0.734
16 to 17	60 (17.1)		31 (15.7)	29 (18.8)	
18 to 20	187 (53.1)		107 (54.0)	80 (52.0)	
21 to 24	105 (29.8)		60 (30.3)	45 (29.2)	
Sexual orientation		351			0.533
Heterosexual	327 (93.2)		183 (92.4)	144 (94.1)	
LGBTQ^a^	24 (6.8)		15 (7.6)	9 (5.9)	
Currently in school		351			0.615
Yes	232 (66.1)		128 (65.0)	104 (67.5)	
No	119 (33.9)		69 (35.0)	50 (32.5)	
Food insecurity^b^		347			0.989
Low	257 (74.1)		143 (74.1)	114 (74.0)	
High	90 (25.9)		50 (25.9)	40 (26.0)	
Housing		352			0.006
Formal	255 (72.4)		132 (66.7)	123 (79.9)	
Informal^c^	97 (27.6)		66 (33.3)	31 (20.1)	
Monthly personal income^d^		352			0.008
< 400 ZAR	93 (26.4)		43 (21.7)	50 (32.5)	
401–1600 ZAR	174 (49.4)		112 (56.6)	62 (40.3)	
1601+ ZAR	85 (24.2)		43 (21.7)	42 (27.3)	
Sexual history and socio-structural variables					
Self-perceived HIV risk		347			0.940
None/low risk	193 (55.6)		107 (55.4)	86 (55.8)	
Medium/high risk	154 (44.4)		86 (44.6)	68 (44.2)	
≥2 Sexual partners in P6m^e^		352			< 0.001
Yes	111 (31.5)		41 (20.7)	70 (45.5)	
No	241 (68.5)		157 (79.3)	84 (54.5)	
Condom use in P6m		343			< 0.001
Consistent (“always”)	111 (32.4)		37 (19.4)	74 (48.7)	
Inconsistent (“sometimes”/“never”)	232 (67.6)		154 (80.6)	78 (51.3)	
Age-disparate sex partner in P6m^f^		340			< 0.001
Yes	85 (25.0)		68 (34.9)	18 (12.4)	
No	255 (75.0)		127 (65.1)	127 (87.6)	
Ever had transactional sex^g^		347			< 0.001
Yes	49 (14.1)		14 (7.2)	35 (22.9)	
No	298 (85.9)		180 (92.8)	118 (77.1)	
Ever been/made pregnant		352			< 0.001
Yes	118 (33.5)		93 (47.0)	25 (16.2)	
No	234 (66.5)		105 (53.0)	129 (83.8)	
Ever experienced physical/sexual violence		347			0.001
Yes	59 (17.0)		45 (23.3)	14 (9.1)	
No	288 (83.0)		148 (76.7)	140 (90.9)	

Notes: ^a^ LGBTQ refers to individuals who identify their sexual orientation as *Lesbian, Gay, Bisexual, Transgender, or Queer*; ^b^ Food insecurity was defined as high if the participant or a household member had ever slept hungry, had no food to eat because of lack of money, or ever went a day and night without eating; ^c^ Informal housing includes RDP housing (government subsidized housing), shacks, or hostels; ^d^ ZAR = South African Rand, with 400 ZAR = $25 USD and 1601 ZAR = $100 USD using the May 30, 2016 currency exchange rate of 1 ZAR = 0.063 USD; ^e^ P6m refers to the 6 months prior to interview; ^f^ Sexual partner is ≥5 years older or younger than participant; ^g^ Transactional sex was classified as ever having sex with someone in exchange for something (given or received)

### GTI symptoms and associated syndromes

Among females, 16.2% (32/198) reported ≥1 GTI symptom. The most commonly reported symptoms were vaginal discharge (7.1%) and vaginal itching or irritation (4.5%). By syndrome, 10.6% of females reported symptoms of VDS, 6.6% of LAP, and 2.0% of GUD. Among males, 0.7% (1/154) reported symptoms compatible with MUS (urethral discharge); no other STI syndromes were described in males (Table 2).

**Table 2:** Sex-stratified prevalence of genital tract infection (GTI) symptoms and associated syndromes reported by sexually experienced, non-pregnant South African youth (aged 16–24 years) enrolled in the AYAZAZI study, overall and by site (*n* = 352)

	Durban	Soweto	Whole cohort
	Females *n* = 96 Males *n* = 83	Females *n* = 102 Males *n* = 71	Females *n* = 198 Males *n* = 154
	*n* (%)	*n* (%)	*n* (%)
Female-specific symptoms:			
Vaginal discharge	6 (6.3)	8 (7.8)	14 (7.1)
Dysuria	0 (0)	4 (3.9)	4 (2.0)
Vaginal Itching or irritation	4 (4.2)	5 (4.9)	9 (4.5)
Vaginal redness or swelling	0 (0)	1 (1.0)	1 (0.5)
Lower abdominal or groin pain (with/without vaginal discharge)	1 (1.0)	4 (3.9)	5 (2.5)
Dyspareunia	0 (0)	2 (2.0)	2 (1.0)
Post-coital bleeding or metrorrhagia	2 (2.1)	4 (3.9)	6 (3.0)
Vaginal sores or skin changes	3 (3.1)	1 (1.0)	4 (2.0)
None of the above	82 (85.4)	82 (80.4)	164 (82.8)
≥ 1 symptom	12 (12.5)	20 (19.6)	32 (16.2)
Female-specific syndromes:			
Vaginal Discharge Syndrome (VDS)	8 (8.3)	13 (12.8)	21 (10.6)
Lower Abdominal Pain (LAP)	3 (3.1)	10 (9.8)	13 (6.6)
Genital Ulcer Disease (GUD)	3 (3.1)	1 (1.0)	4 (2.0)
Male-specific symptoms:			
Sores or skin changes around penis or scrotum	0 (0)	0 (0)	0 (0)
Urethral discharge	0 (0)	1 (1.4)	1 (0.7)
Dysuria	0 (0)	0 (0)	0 (0)
Pain, swelling or redness of scrotum	0 (0)	0 (0)	0 (0)
Pain in testes	0 (0)	0 (0)	0 (0)
Soreness or itching around foreskin (if uncircumcised)	0 (0)	0 (0)	0 (0)
Hot tender swelling with redness	0 (0)	0 (0)	0 (0)
None of the above	83 (100.0)	70 (98.6)	153 (99.4)
≥ 1 symptom	0 (0)	1 (1.4)	1 (0.7)
Male-specific syndromes:			
Genital Ulcer Disease (GUD)	0 (0)	0 (0)	0 (0)
Male Urethritis (MUS)	0 (0)	1 (1.4)	1 (0.7)
Scrotal Swelling (SSW)	0 (0)	0 (0)	0 (0)
Balanitis	0 (0)	0 (0)	0 (0)
Bubo	0 (0)	0 (0)	0 (0)

Notes: GTI symptoms and syndromes are classified based on WHO and South African STI management guidelines (WHO 2004, South African Department of Health 2015)

### Laboratory-confirmed prevalence of GTIs

Laboratory tests identified ≥1 GTI in 44.0% of study participants, including 70.2% of females and 10.4% of males (p < 0.001) (Table 3).

**Table 3:** Sex-stratified prevalence of laboratory-confirmed genital tract infections (GTIs) among sexually experienced, non-pregnant South African youth (aged 16–24 years) enrolled in the AYAZAZI study, overall and by study site (*n* = 352)

Laboratory-confirmed infections	Durban			Soweto			Whole cohort		
	Female *n* = 96	Male *n* = 83	Total *n* = 179	Female *n* = 102	Male *n* = 71	Total *n* = 173	Female *n* = 198	Male *n* = 154	Total *n* = 352
≥ 1 GTI (excluding HIV)	66 (68.8)	10 (12.0)	76 (42.5)	73 (71.6)	6 (8.5)	79 (45.7)	139 (70.2)^*^	16 (10.4)^*^	155 (44.0)
Non-ulcerative STIs									
*C. trachomatis* (CT)	14 (14.6)	7 (8.4)	21 (11.7)	22 (21.6)	5 (7.0)	27 (15.6)	36 (18.2)^*^	12 (7.8)^*^	48 (13.6)
*N. gonorrhoeae* (NG)	5 (5.2)	2 (2.4)	7 (3.9)	9 (8.8)	0 (0)	9 (5.2)	14 (7.1)^*^	2 (1.3)^*^	16 (4.6)
*M. genitalium* (MG)	5 (5.2)	3 (3.6)	8 (4.5)	14 (13.7)	2 (2.8)	16 (9.3)	19 (9.6)^*^	5 (3.3)^*^	24 (6.8)
*T. vaginalis* (TV)	3 (3.1)	0 (0)	3 (1.7)	13 (12.8)	1 (1.4)	14 (8.1)	16 (8.1)^*^	1 (0.7)^*^	17 (4.8)
≥ 1 of CT/NG/MG	22 (22.9)	10 (12.0)	32 (17.9)	38 (37.3)	5 (7.0)	43 (24.9)	60 (30.3)^*^	15 (9.7)^*^	75 (21.3)
≥ 1 non-ulcerative STI	24 (25.0)	10 (12.0)	34 (19.0)	45 (44.1)	6 (8.5)	51 (29.5)	69 (34.8)^*^	16 (10.4)^*^	85 (24.1)
Female-only GTIs									
Bacterial Vaginosis (BV)	52 (54.2)			53 (52.0)			105 (53.0)		
*C. albicans* (CA)	7 (7.3)			12 (11.8)			19 (9.6)		
Co-infection									
≥ 2 non-ulcerative STIs	3 (3.1)	2 (2.4)	5 (2.8)	11 (10.8)	2 (2.8)	13 (7.5)	14 (7.0)^*^	4 (2.6)^*^	18 (5.0)
≥ 1 non-ulcerative STI & ≥1 GTI	16 (16.7)			34 (33.3)			50 (25.3)		
*Among those with ≥ 1 non-ulcerative STI*									
≥ 2 non-ulcerative STIs	3 (12.5)	2 (20.0)	5 (14.7)	11 (24.4)	2 (33.3)	13 (25.5)	14 (20.3)	4 (25.0)	18 (21.2)
≥ 1 non-ulcerative STI & ≥1 GTI	16 (66.7)			34 (75.6)			50 (72.5)		
Ulcerative STIs (among those with genital ulcers)^a^	*n* = 1	*n* = 0	*n* = 1	*n* = 1	*n* = 1	*n* = 2	*n* = 2	*n* = 1	*n* = 3
HSV-2	1 (100.0)	0 (0)	1 (100.0)	0 (0)	0 (0)	0 (0)	1 (50.0)	0 (0)	1 (33.3)
*T. pallidum*	0 (0)	0 (0)	0 (0)	0 (0)	0 (0)	0 (0)	0 (0)	0 (0)	0 (0)
*H. ducreyi*	0 (0)	0 (0)	0 (0)	0 (0)	0 (0)	0 (0)	0 (0)	0 (0)	0 (0)
LGV	0 (0)	0 (0)	0 (0)	0 (0)	0 (0)	0 (0)	0 (0)	0 (0)	0 (0)
HIV^b^	9 (9.4)	2 (2.4)	11 (6.2)	1 (1.0)	1 (1.4)	2 (1.2)	10 (5.1)	3 (2.0)	13 (3.7)

Notes: *Statistically significant difference (*p* < 0.05) between prevalence among females vs males; ^a^Prevalence of ulcerative STIs restricted to the n = 3 participants who had a genital ulcer identified during physical exams, which was swabbed and tested using multiplex NAAT testing; ^b^As per the study inclusion criteria, all participants reported being HIV-negative or unknown HIV status at enrollment

Among female participants, over one-third (34.8%; *n* = 69/198) were infected with at least one non-ulcerative STI, including 18.2% with CT, 7.1% with NG, 9.6% with MG, and 8.1% with TV. Of those infected with ≥1 one non-ulcerative STI (*n* = 69), one-fifth (20.3%; *n* = 14) were co-infected with other STI pathogens while nearly three-quarters (72.5%; *n* = 50) were co-infected with a GTI. By site, the prevalence of non-ulcerative STIs was higher among females in Soweto compared with Durban (44.1% and 25.0% respectively, *p* = 0.008). Of two young women with genital ulcers, one case of herpes was confirmed and there were no cases of laboratory-proven syphilis. Just over half (53.0%) of all females tested positive for BV, with a further 11 females having intermediate flora (5.6%). Prevalence of CA was 9.6%. One-quarter (25.3%) of females were co-infected with a non-ulcerative STI and BV or CA. Ten female participants (5.1%) were newly diagnosed with HIV (median CD4 = 301 cells/mm^3^ [IQR: 197–392], with a significantly higher baseline HIV prevalence in Durban than Soweto (9.4% and 1.0%, respectively; *p* = 0.008). Overall, 90.0% (9/10) of HIV-positive females were co-infected with BV, while none had an STI.

Among male participants, one-tenth (10.4%; *n* = 16/154) were infected with at least one non-ulcerative STI, including 7.8% with CT, 1.3% with NG, 3.3% with MG, and 0.7% with TV. Of those infected with an MUS-associated STI, one-quarter (25.0%) were co-infected. No STI pathogen was detected in the single male participant with genital ulcers. Three male participants (2.0%) were newly diagnosed with HIV (median CD4 = 102 cells/mm^3^ IQR: 85–119), with a non-statistically significant higher HIV prevalence in Durban compared to Soweto (2.4% and 1.4%, respectively, *p* = 1.00). No HIV-positive males were co-infected with an STI.

### Sensitivity analysis

Overall, only 22.2% of females and 0% of males with a confirmed GTI reported at least one GTI symptom. In other words, 77.8% of females and 100% of males with laboratory-diagnosed GTIs reported no accompanying symptoms or were asymptomatic (Table 4).

**Table 4:** Sex-stratified sensitivity analyses of syndromic screening for genital tract infections (GTIs) among sexually experienced, non-pregnant South African youth (aged 16–24 years) enrolled in the AYAZAZI study (*n* = 352)

Syndrome	Symptomatic	Laboratory test (gold standard)		Total	Sensitivity (%) [95% CI]	Specificity (%) [95% CI]	PPV (%) [95% CI]	NPV (%) [95% CI]
		Positive	Negative					
*Females (n = 198)*								
Vaginal discharge (VDS)^a^	Yes	19	2	21	13.7	96.6	90.5	32.2
	No	120	57	177	[8.4–20.5]	[88.2–99.6]	[69.6–98.8]	[25.4–39.6]
with cervical STIs as causative agents^b^	Yes	7	14	21	11.7	89.9	33.3	70.1
	No	53	124	177	[4.8–22.6]	[83.6–94.3]	[14.6–57.0]	[62.7–76.7]
with vaginal STIs as causative agents^c^	Yes	18	3	21	14.8	96.1	85.7	41.2
	No	104	73	177	[9.0–22.3]	[88.9–99.2]	[63.7–97.0]	[33.9–48.9]
with only BV as a causative agent	Yes	14	7	21	13.3	92.5	66.7	48.6
	No	91	86	177	[7.5–21.4]	[85.1–96.9]	[43.0–85.4]	[41.0–56.2]
Lower abdominal pain (LAP)^d^	Yes	4	9	13	7.0	93.4	30.8	70.6
	No	53	127	180	[1.9–17.0]	[87.8–97.0]	[9.1–61.4]	[63.3–77.1]
*Males (n = 154)*								
Male urethritis (MUS)^e^	Yes	0	1	1	0.0	99.3	0.0	89.5
	No	16	137	153	[0–20.6]^g^	[96.0–100.0]	[0–97.5]^g^	[83.6–93.9]
Scrotal Swelling (SSW)^f^	Yes	0	0	0	0.0	100.0	N/A	91.6
	No	13	141	154	[0–24.7]^g^	[97.4–100.0]^g^	[N/A]	[86.0–95.4]

Notes: GTIs included as the gold standard for VDS, LAP, MUS and SSW include (note: no sensitivity analysis was performed for GUD given testing via genital swabs and NAAT:

^a^Any STI/GTI with VDS as a syndrome: *N.gonorrhoeae, C.trachomatis, M.genitalium* (cervical infection), *T.vaginalis*, BV, or *C. albicans* (vaginal infection);

^b^Any cervical STI with VDS as a syndrome: *N.gonorrhoeae, C.trachomatis, M.genitalium*;

^c^Any vaginal STI with VDS as a syndrome: *T.vaginalis*, BV, *C. albicans;*

^d^Any STI/GTI with LAP as a syndrome: *N.gonorrhoeae, C.trachomatis, M.genitalium*

^e^Any STI with MUS as syndrome: *N.gonorrhoeae, C.trachomatis, M.genitalium, T.vaginalis;*

^f^Any STI with SSW as syndrome: *N.gonorrhoeae, C.trachomatis;*

^g^1-sided 97.5% CI

#### Females

Sensitivity analyses revealed that VDS symptomology poorly indicated infection with associated pathological organisms (i.e., NG, CT, MG, TV, BV, or CA). Overall, 19/21 reporting VDS symptoms were infected with a VDS-associated GTI; however, of 139 females with laboratory-confirmed infections with a VDS-associated GTI, just 19 reported symptoms (sensitivity 13.7%, specificity 96.6%, PPV 90.5%, NPV 32.2%). When restricted to cervical STIs as the causative agents of VDS, sensitivity, specificity and PPV all decreased to 11.7%, 89.9%, and 33.3% (respectively), while NPV increased to 70.1%. When restricted to vaginal STIs as the causative agents of VDS or only BV, performance measures were also poor (for BV: sensitivity 13.3%, specificity 92.5, PPV 66.7%, NPV 48.6%).

Similarly, LAP symptomology poorly indicated infection with associated pathogens (i.e., NG, CT, MG), with both poor sensitivity and poor PPV. Overall, only 4/13 females reporting LAP symptoms were infected with a LAP-associated GTI; while of 57 females with confirmed infection with a LAP-associated GTI, just 4 reported symptoms (sensitivity 7.0%, specificity 93.4%, PPV 30.8%, NPV 70.6%).

#### Males

No MUS-associated pathogen (i.e., NG, CT, MG, and/or TV) was detected in the one male reporting urethral discharge. Of 16 males truly infected with a MUS-associated STI, none reported symptoms (sensitivity 0%, specificity 99.3%, PPV 0%, NPV 89.5%).

## Discussion

GTIs, including asymptomatic infections, induce genital tract inflammation among young women, increasing HIV acquisition risk [14]. We found a high and frequently asymptomatic burden of GTI infection among South African youth, with significant differences by sex. While only 16% of young women and < 1% of young men reported GTI-related symptoms, laboratory tests identified at least one GTI in 70% of females and 10% of males. Syndromic GTI management revealed very poor sensitivity. The high burden of asymptomatic GTIs contributes to a silent GTI epidemic in youth, which requires additional attention, innovations, and resources to control. We strongly recommend policy changes to replace syndromic management with innovative public health approaches, inclusive of widespread diagnostic testing to reduce GTI burden and HIV-associated risks among youth.

The high GTI prevalence observed here is particularly notable, given that participants are young (16–24 years of age), primarily recruited from community settings, and were not enrolled due to high GTI risk. Nonetheless, over one-third of young women had at least one non-ulcerative STI and half had laboratory-detected BV. The prevalence of each non-ulcerative STI (CT: 18.2%; NG: 7.1%; MG: 9.6%; TV: 8.1%) was higher or similar to the prevalence observed among older women (18–40 years) presenting for STI care in Durban (CT: 18.4%; NG: 5.2%; MG: not assessed; TV: 3.0%) [16], high HIV-risk women enrolling in the CAPRISA 002 Acute HIV Infection study in Durban (overall STI prevalence = 25.3%; CT: 4.2%; NG: 5.4%; MG: 1.2%; TV: 20.3%) [17], women enrolled in three large clinical trials in Durban (STI prevalence = 13%) [18], “high HIV risk” Tanzanian women (CT: 12%; NG: 4%; MG: not assessed; TV: 19%) [19], and women attending antenatal care in Kenya (CT: 14.9%; NG: 1.0%; MG: not assessed; TV: 7.4%) [20]. The exception to this pattern was TV, which was lower in female youth in our study relative to older women in both the Tanzanian and South African studies. Such patterns are consistent with known biological reasons for increases in TV prevalence with age (i.e., protozoal accumulation over time). The high BV prevalence (53.0%) mirrors levels reported in other South African studies [4, 17, 18, 21, 22].

Fewer (10%) young men in our study had a laboratory-confirmed non-ulcerative STI compared to women. STI prevalence among men was driven largely by CT (7.8%). Our findings are consistent with the limited available data regarding STI prevalence for young and older heterosexual men in South Africa [23, 24].

We observed a 3.7% prevalence of HIV sero-positivity at baseline, despite study eligibility criteria aimed at enrolling HIV-negative participants. Moreover, low median CD4 cell counts at HIV diagnosis signal advanced HIV disease progression. HIV prevalence was higher among Durban participants compared to Soweto, consistent with findings from surveillance data [1]. Given the known synergistic relationship between HIV and STI co-infection, the higher prevalence of non-ulcerative STIs but lower prevalence of HIV in Soweto is notable.

Consistent with previous reports, syndromic management was unable to detect the majority of GTIs due to their asymptomatic nature [17, 25]. Without laboratory testing, approximately 80% of prevalent GTIs among young women and 100% among young men in our study would have remained untreated. Poor sexual health knowledge and a lack of familiarity with GTI symptoms among youth may have exacerbated under-reporting of symptoms [26–28]. Both VDS and LAP had poor sensitivity (13.7% and 7.0% respectively). This is a well-documented limitation to the VDS algorithm, especially for cervical infection [17, 21, 29]. VDS had a high PPV (90.5%) indicating that most women who reported VDS-related symptoms did have a laboratory-confirmed GTI and would have been appropriately treated under syndromic management. That is, syndromic management performed appropriately in its role of detecting and managing symptomatic GTIs. However, the PPV for LAP was poor (30.8%) highlighting that a majority of young women who reported LAP-associated symptoms did not have a laboratory-confirmed GTI and may have been inappropriately treated under syndromic management guidelines. Insufficient symptomatic infection in young men in our study precluded robust sensitivity analyses of syndromic management; no men with an STI reported symptoms and no men who reported symptoms had an STI.

There are important advantages to syndromic management on a national scale; the primary being cost, and a second being that it requires a single visit, decreasing the likelihood of treatment loss-to-follow-up at a second visit, typically required with diagnostic testing [9]. These advantages must be considered against the performance of syndromic approaches for high-priority populations. Our findings suggest that in South Africa, both male and female youth have a high burden of undetected GTIs. Reliance on a syndromic management approach will continue to yield low detection of prevalent GTIs and thus poor GTI control.

Our findings suggest a need to evolve sexual health services to benefit youth. Although a test-and-treat approach to GTIs has historically posed financial and logistical challenges, recent innovations in affordable real-time STI testing, such as point-of-care (POC) diagnostics, warrant further investigation for wide-scale implementation [16, 30]. By providing immediate testing results and facilitating treatment without delay, POC tests can help reduce secondary transmission and the consequences of infection progression [31]. POC testing also presents an opportunity to screen outside of traditional clinic settings, which young people infrequently access [32], and move testing into community-based and youth-friendly settings that offer comprehensive HIV and GTI services. POC testing quality is growing with improving technology [33], with good performance across several prevalent STIs, including CT, NG, TV, and syphilis [34, 35]. Recent work also suggests that POC testing for STIs is acceptable to providers [33] and to young women and their partners [16]. Additional studies are warranted to assess the cost-effectiveness of POC STI testing for youth in low- and middle-income countries.

GTI control will not be solved by diagnostic innovation alone. In addition to GTI burden, and consistent with previous studies [1], youth in our study report high behavioral, social, and structural vulnerability for GTI and HIV acquisition. Notably, over half of adolescent and young adults in this HIV hyper-endemic setting self-perceived themselves as being at low or no risk of HIV acquisition. These findings underscore the need for efforts to move beyond individual-level risk factors for promoting healthy sexuality for young people, and address the social and structural risk environments, such as gender and power inequity in relationships, coercive sex, economic inequity, violence, and poverty, which shape how young people navigate their sexual lives.

This study has limitations. First, HSV-2 and syphilis prevalence are likely to be under-estimated as the study protocol specified NAAT testing of genital ulcerative lesions only. Future work should incorporate serological testing to assess the true prevalence of both HSV-2 and syphilis in youth. Second, not all GTIs were tested (for example, anorectal samples were not taken), thus the reported GTI prevalence may be an underestimate. Third, participants were asked directly about GTI symptoms and their genital areas carefully examined by a study nurse with expertise in youth-focused care delivery, and may have been more willing to report symptoms or undergo genital examination. Thus, our assessment of the sensitivity and PPV of syndromic management of GTIs among youth, although very low, is likely higher than would be observed in a public care setting.

## Conclusion

Maintaining the status quo of syndromic GTI management for youth is not acceptable. We observed a substantial burden of subclinical GTI infection alongside HIV risk behaviors and low risk-perception for HIV acquisition among South African youth. Sensitivity of symptom-based reporting was poor and offered no value in identifying the highly prevalent asymptomatic GTI infections. Syndromic management does not meet the needs of young people in this and similar settings and leaves them at increased risk of HIV acquisition. In the context of evolving and less costly GTI diagnostic capabilities, exploring new approaches to implementing youth-focused GTI services is warranted.

## Additional file

Additional file 1: **Table S1.** Genital Tract Infection (GTI) symptoms and associated syndromes assessed for male and female participants (aged 16–24 years) enrolled in the AYAZAZI study. (DOCX 14 kb)

## Abbreviations

ART: Antiretroviral therapy
BV: Bacterial Vaginosis
CT: *Chlamydia trachomatis*
ELISA: Enzyme-linked immunosorbent assay
GTI: Genital tract infection
GUD: Genital Ulcer Disease
HCG: Human Chorionic Gonadotropin
HIV: Human Immunodeficiency Virus
HSV-2: Herpes simplex virus type 2
IQR: Interquartile Range
LAP: Lower Abdominal Pain
MG: *Mycoplasma genitalium*
MRU: MatCH Research Unit
MUS: Male Urethritis Syndrome
NAAT: Nucleic Acid Amplification Test
NG: *Neisseria gonorrhoeae*
NPV: Negative Predictive Value
PHRU: Perinatal HIV Research Unit
POC: Point-of-care
PPV: Positive Predictive Value
SSW: Scrotal Swelling
STI: Sexually Transmitted Infection
TV: *Trichomonas vaginalis*
VDS: Vaginal Discharge Syndrome
ZAR: South African Rand
