# Supplementary material for: A high burden of asymptomatic genital tract infections undermines the syndromic management approach among adolescents and young adults in South Africa: implications for HIV prevention efforts
Source: BMC Infect Dis. 2018 Oct 3;18:499. doi: 10.1186/s12879-018-3380-6 (PMC6171143; doi:10.1186/s12879-018-3380-6)
Supplement: Supplementary file 1 — Table S1. Genital Tract Infection (GTI) symptoms and associated syndromes assessed for male and female participants (aged 16–24 years) enrolled in the AYAZAZI study. (DOCX 14 kb) [file 12879_2018_3380_MOESM1_ESM.docx]

**Table S1. Genital Tract Infection (GTI) symptoms and associated syndromes assessed for male and female participants (aged 16-24 years) enrolled in the AYAZAZI study**

| **Participant Sex** | **Syndromes** | **Symptoms** |
| --- | --- | --- |
| Female | Vaginal Discharge Syndrome (VDS) | ***Any of:*** Vaginal discharge, dysuria, vaginal itching/irritation, vaginal redness or swelling |
| Female | Lower Abdominal Pain (LAP) | ***Any of:*** Lower abdominal/groin pain (with or without vaginal discharge), post-coital bleeding or metrorrhagia. |
| Female | Genital Ulcer Disease (GUD) | Vaginal sores or skin changes around vagina |
| Male | Genital Ulcer Disease (GUD) | Sores or skin changes around penis or scrotum |
| Male | Male Urethritis Syndrome (MUS) | ***Any of:*** Urethral discharge, dysuria |
| Male | Scrotal Swelling (SSW) | ***Any of:*** Pain, swelling or redness of scrotum, pain in testes |
| Male | Balanitis | Soreness or itching around the foreskin |
| Male | Bubo | Hot tender swelling with redness |
